# Extranodal NK/T-Cell Lymphoma, Nasal Type, Presenting as a Breast Mass

**DOI:** 10.7759/cureus.408

**Published:** 2015-12-15

**Authors:** Ahmad Rahal, Pavan S Reddy, Carmelita Alvares

**Affiliations:** 1 Internal Medicine, University of Kansas School of Medicine-Wichita; 2 Pathology, University of Kansas School of Medicine-Wichita

**Keywords:** breast ca, extra-nodal nk/t-cell lymphoma, smile regimen, epstein-barr virus (ebv)

## Abstract

Extranodal natural killer/T-cell lymphoma, nasal type, is a rare type of non-Hodgkin cell lymphoma endemic to East Asia and parts of Central and South America. In most cases, it is driven by Epstein-Barr virus infections, with a broad range of morphologic appearances, frequent necrosis, and angioinvasion. It is designated as NK/T reflecting uncertainty in its cellular origins. These tumors usually arise in the nasal region, typically presenting with symptoms of nasal obstruction, epistaxis, and/or a destructive mass involving the nose, sinuses, or palate. The treatment of patients with extranodal NK/T-cell lymphoma, nasal type, is largely determined by the extent of disease. Localized disease is usually treated with radiation and chemotherapy. The disseminated disease requires combination chemotherapy. This report describes the case of a 30-year-old Caucasian female presenting with a left breast mass of two months duration. Excisional biopsy was done, and the pathological exam confirmed the diagnosis of extranodal NK/T-cell lymphoma, nasal type. Our patient received a systemic combination chemotherapy with steroid (dexamethasone), methotrexate, ifosfamide, L-asparaginase, and etoposide (SMILE) regimen, resulting in a complete clinical and radiological remission. On the basis of our review of the literature, extranodal NK/T non-Hodgkin cell lymphoma, nasal type, presenting as a breast mass is very rare and very uncommon in the United States. Awareness of this occurrence may be valuable as this case may be a forerunner of additional similar cases developing in the future.

## Introduction

Extranodal NK/T-cell lymphoma, nasal type (ENKTL) is a rare extranodal lymphoma driven by Epstein-Barr virus (EBV) infections, most commonly involving the nasal area [1]. Occasionally, patients with ENKTL present with only extranasal sites of disease, most often skin, lung, and gastrointestinal tract, with an involvement of the breast being rare. ENKTL has a greater prevalence in Asian and Latin American regions and is rare in the United States [2]. This neoplasm can have an NK-cell or a T-cell immunophenotype and is virtually always positive for Epstein-Barr virus (EBV), suggesting that EBV plays a major pathogenic role. The majority of patients present with nasal obstruction, sinusitis, and epistaxis due to a destructive mass involving the midline facial tissues. The treatment of patients with extranodal NK/T-cell lymphoma, nasal type, is largely determined by the extent of disease. Localized disease is usually treated with radiation and chemotherapy. The disseminated disease requires combination chemotherapy. In this study, we present a rare case of ENKTL presenting as a breast mass in a 30-year-old Caucasian female in the United States.

## Case presentation

**Figure 1:**
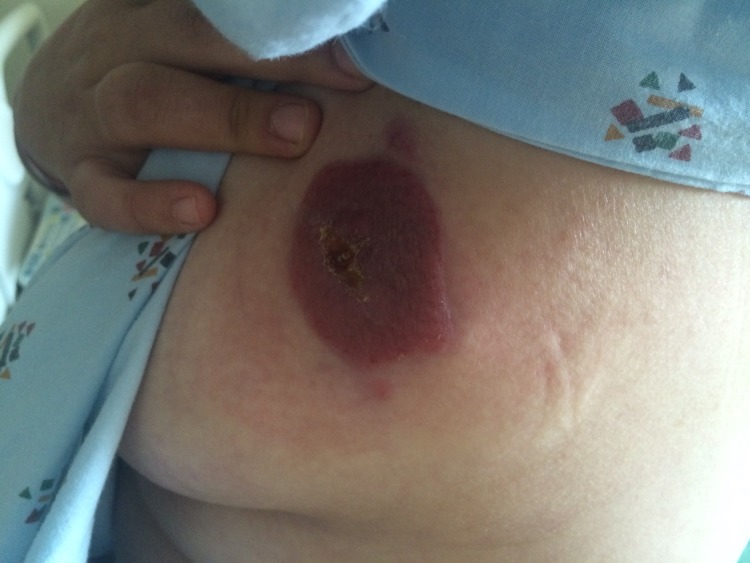
Left breast lesion before treatment

A 30-year-old Caucasian female patient with a past medical history significant for arthritis presented with a two-month history of a painless, non-healing left breast mass. The patient denied any fevers, chills, night sweats, weight loss, nipple discharge, or family history of cancer. On physical exam, there was an erythematous, thickened, well-circumscribed lesion on the lateral aspect of the left breast roughly measuring 4 x 4 cm in size with no active drainage (Figure 1). No palpable hepatosplenomegaly, supraclavicular, axillary, or inguinal lymphadenopathy was found. Informed patient consent was obtained. Excisional biopsy of the left breast mass was done and the pathological exam, including microscopic evaluation, and flow cytometry, showed medium-sized mononuclear cells with vesicular nuclei. There were brisk mitotic activity and small zones of necrosis. The mononuclear infiltrate showed diffuse positivity for CD45, CD3, and CD56. TIA-1 was strongly positive within tumor cells, indicative of a cytotoxic phenotype. EBV in situ hybridization was also strongly positive within the tumor cells. The pathological findings confirmed the diagnosis of extranodal NK/T non-Hodgkin cell lymphoma, nasal type.

**Figure 2:**
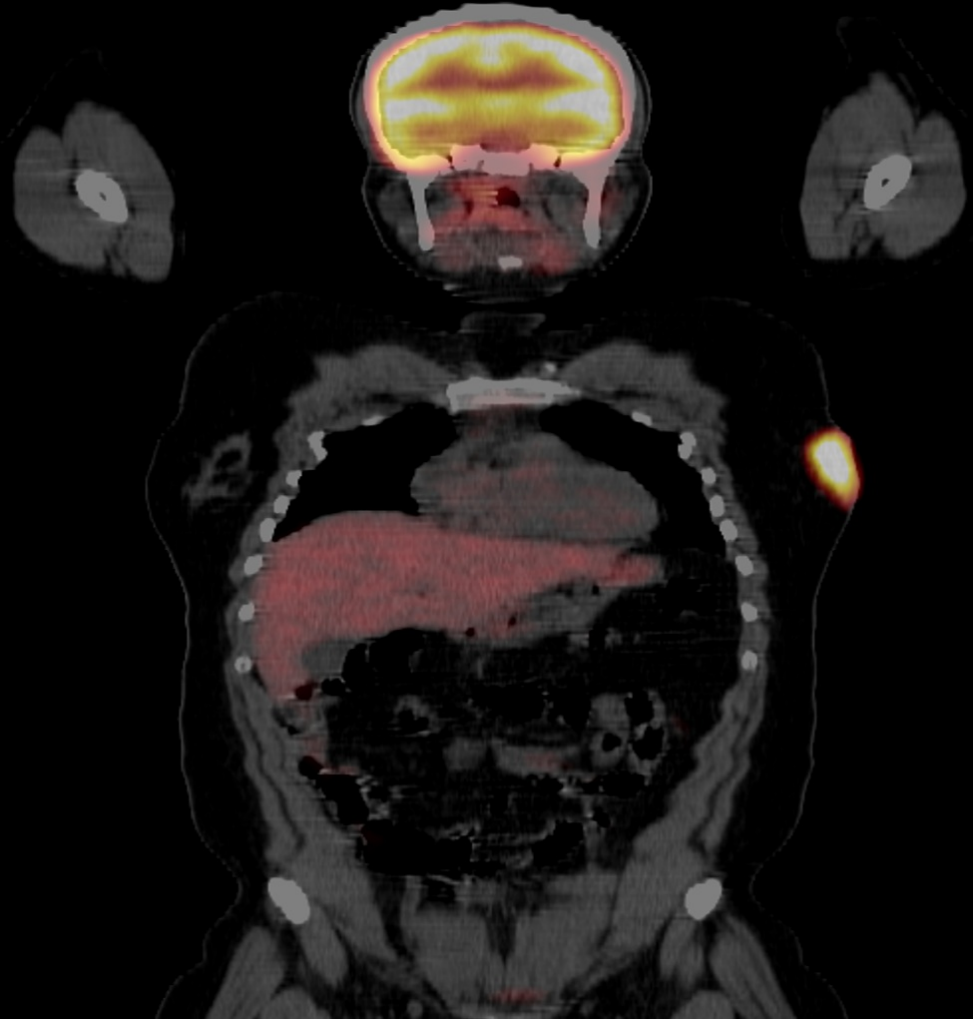
Baseline PET/CT scan showing lateral left breast uptake

A complete staging workup was initiated. Laboratory studies, including a complete blood count with differential, chemistries with liver and renal function, electrolytes, lactate dehydrogenase, hepatitis B virus, human immunodeficiency virus, and uric acid, were unremarkable. A positron emission tomography (PET)/CT scan showed corresponding hypermetabolic uptake in the left breast (Figure 2) and left nasal cavity.

**Figure 3:**
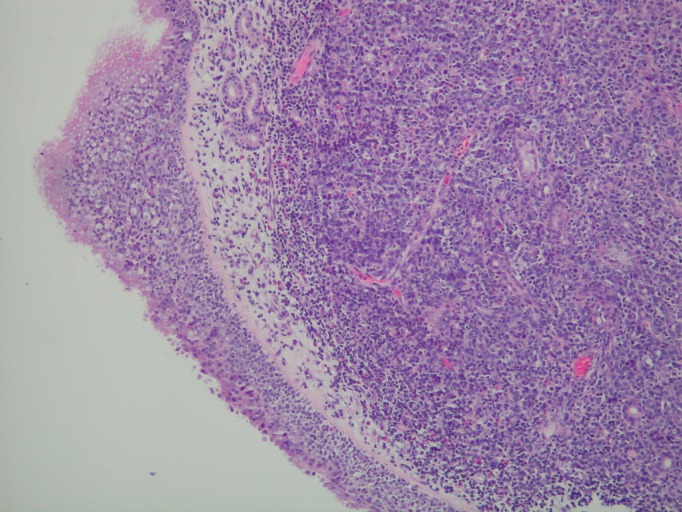
Sinonasal mucosa with underlying tumor infiltrate (H&E, 100x magnification)

**Figure 4:**
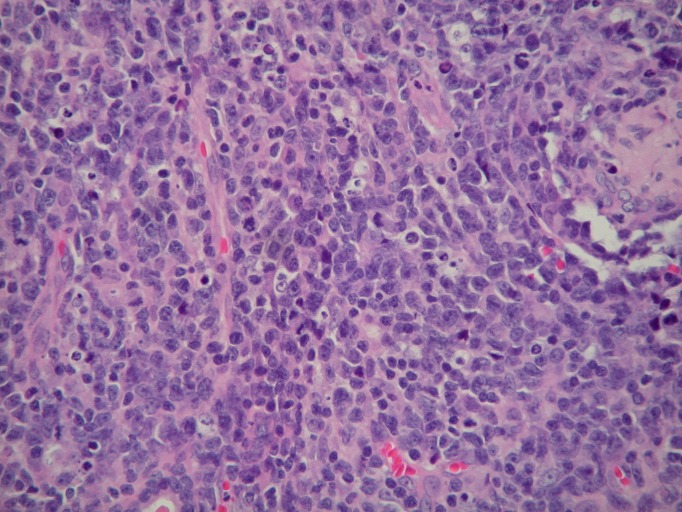
Microscopic appearance of left nasal lesion Tumor comprised of medium to large size mononuclear cells with vesicular nuclei, single to multiple inconspicuous nucleoli, scant to moderate pale eosinophilic cytoplasm, frequent mitoses and angiodestructive pattern, and sprinkling of small lymphocytes and a few eosinophils (H&E, 400x magnification).

Nasal endoscopy revealed a left inferior turbinate mass. The pathological examination, including microscopic evaluation and flow cytometry, showed a diffuse and angiodestructive invasive pattern, admixed with frequent mitoses and apoptosis (Figures 3-4). Immunophenotypic analysis of the left inferior turbinate mass revealed a predominant population of abnormal lymphoid cells positive for CD2, HLA-DR, CD56, and CD38, confirming the diagnosis of NK/T-cell lymphoma.

**Figure 5:**
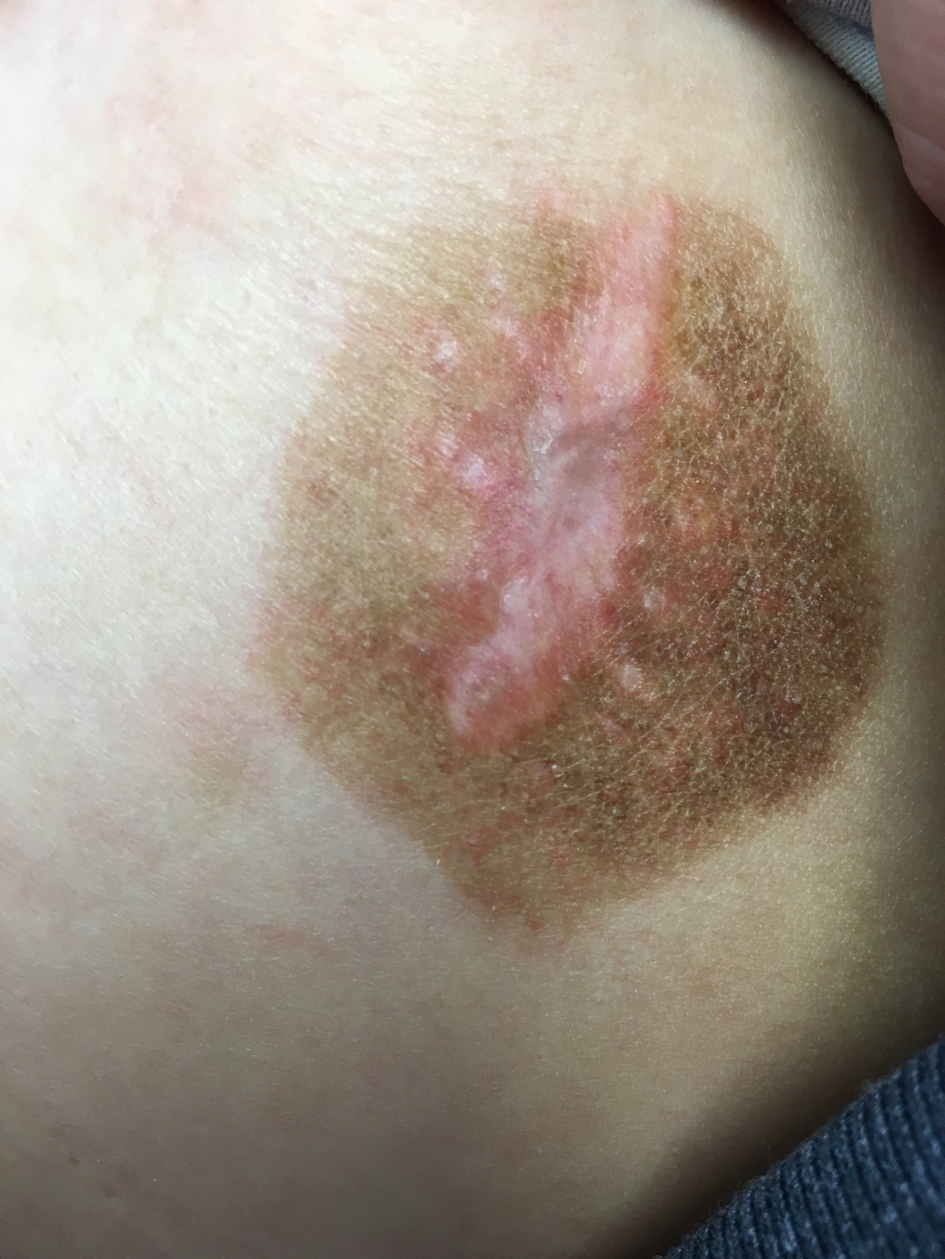
Improvement of left breast lesion after treatment with combined systemic chemotherapy

**Figure 6:**
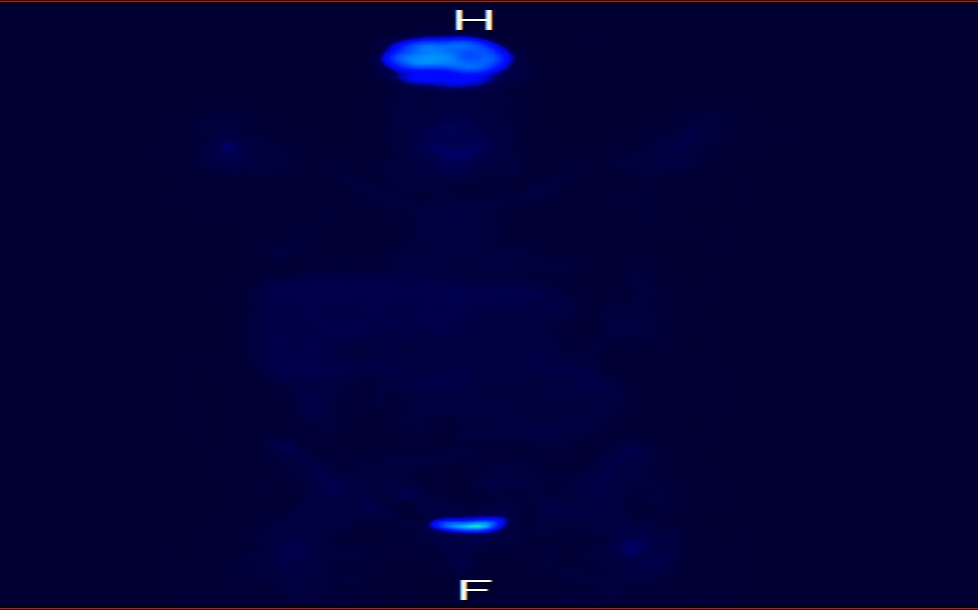
PET/CT scan after therapy with systemic combined chemotherapy showing absence of uptake in the left breast.

Nasopharyngoscopy with nasopharyngeal biopsies was negative for a mass or malignancy. Bone marrow biopsy and aspiration were negative for lymphoma. An echocardiogram showed normal systolic function with an ejection fraction of 55-60%. The patient was started on combination chemotherapy using dexamethasone, methotrexate, ifosfamide, L-asparaginase, and etoposide (SMILE regimen). After four cycles of SMILE chemotherapy, the patient underwent complete clinical and radiological remission (Figures 5-6). After achieving remission, the patient will proceed with consolidation with high-dose chemotherapy and an autologous hematopoietic cell transplantation.

## Discussion

Extranodal NK/T-cell lymphoma, nasal type, is an aggressive extranodal lymphoma of either natural killer (NK) or T-cell lineage. Most of the ENKTL occur in the nasal-paranasal area. The remainder usually involves the skin/soft tissue, testis, gastrointestinal tract, and muscles [1]. The pathogenesis of ENKTL is poorly understood but is related in part to infection of the tumor cells with EBV. The large majority of patients present with localized disease, resulting in symptoms of nasal obstruction, epistaxis, and/or a destructive mass involving the nose, sinuses, or palate [3]. PET/CT scan is useful in staging, as lymphomas are 18-fluorodeoxyglucose-avid.

Most lymphomas that involve the breast, either as a primary disease or as a part of dissemination, are B-cell lymphomas, most often diffuse large B-cell lymphoma and extranodal marginal zone lymphoma of mucosa-associated lymphoid tissue (MALT lymphoma), whereas T-cell lymphomas involving the breast are uncommon [4]. Very few cases of ENKTL involving the breast have been reported. Three of these cases reported by Cho, et al. [5] and by Fréling, et al. [6] were described as involving skin and soft tissue, rather than breast parenchyma. One reported case involved the breast as a part of the disseminated disease in an immunosuppressed woman who had undergone heart transplantation five years earlier [7]. Two cases were also reported in patients that previously received breast implants [8-9].  

The case we report is rare because extranodal NK/T-cell lymphoma of the nasal type rarely involves the breast, and even more rarely arises in the breast in an immunocompetent individual. This case is unusual for other reasons as well. Extranodal NK/T-cell lymphoma of the nasal type is uncommon among whites and in the United States. The prognosis of nasal NK/T-cell lymphoma is extremely poor, especially when other systemic sites are involved. Without treatment, survival is measured in months.

Current treatment strategies include radiotherapy together with chemotherapy for localized disease. In disseminated NK/T-cell lymphomas, systemic combination chemotherapy remains the mainstay of treatment. The role of hematopoietic stem cell transplantation has not been prospectively evaluated. Approximately 30% of patients with localized disease and 80% of patients with disseminated disease at the time of diagnosis will either not achieve remission or will relapse. Our patient achieved complete clinical and radiological remission with SMILE chemotherapy and will now proceed with consolidation with high-dose chemotherapy and autologous hematopoietic cell transplantation.

## Conclusions

In summary, we describe a unique case of extranodal NK/T-cell lymphoma, nasal type, presenting as a breast mass in a Caucasian female from the United States. The best treatment option for patients with Stage IV extranodal NK/T-cell lymphoma, nasal type, is unknown, and patients should be encouraged to participate in clinical trials whenever possible. Several regimens incorporating L-asparaginase, such as the SMILE regimen, have shown promising results as observed in our patient. Awareness of this occurrence may also be valuable as this case may be a forerunner of additional similar cases developing in the future. 
